# Identification and validation of p50 as the cellular target of eriocalyxin B

**DOI:** 10.18632/oncotarget.2461

**Published:** 2014-10-24

**Authors:** Ling-Mei Kong, Xu Deng, Zhi-Li Zuo, Han-Dong Sun, Qin-Shi Zhao, Yan Li

**Affiliations:** ^1^ State Key Laboratory of Phytochemistry and Plant Resources in West China, Kunming Institute of Botany, Chinese Academy of Sciences, Kunming 650201, China; ^2^ Graduate University of the Chinese Academy of Sciences, Beijing 100039, China

**Keywords:** EriB, activity-based probes, p50, NF-kB signaling, apoptosis

## Abstract

As an *ent*-kaurene diterpenoid isolated from *Isodon eriocalyx var. Laxiflora*, Eriocalyxin B (EriB) possesses potent bioactivity of antitumor and anti-autoimmune inflammation, which has been suggested to work through inhibition of NF-kappaB (NF-κB) signaling. However, the direct target of EriB remains elusive. In this study, we showed that EriB induced apoptosis is associated with the inhibition of NF-κB signaling in SMMC-7721 hepatocellular carcinoma cells. With activity-based probe profiling, we identified p50 protein as the direct target of EriB. We showed that cysteine 62 is the critical residue of p50 for EriB binding through the α, β-unsaturated ketones. As the result, EriB selectively blocks the binding between p50 and the response elements, whereas having no effect on the dimerization or the nuclear translocation of p50 and p65. SiRNA mediated knockdown of p50 attenuated the apoptosis induced by EriB in SMMC-7721 cells. Taken together, our studies illustrated that EriB induces cancer cell apoptosis through interfering with the binding between NF-κB and the response elements by targeting the cysteine 62 of p50, which highlights its potential for the development of p50 targeted cancer therapeutic agents.

## INTRODUCTION

**Figure 1:**
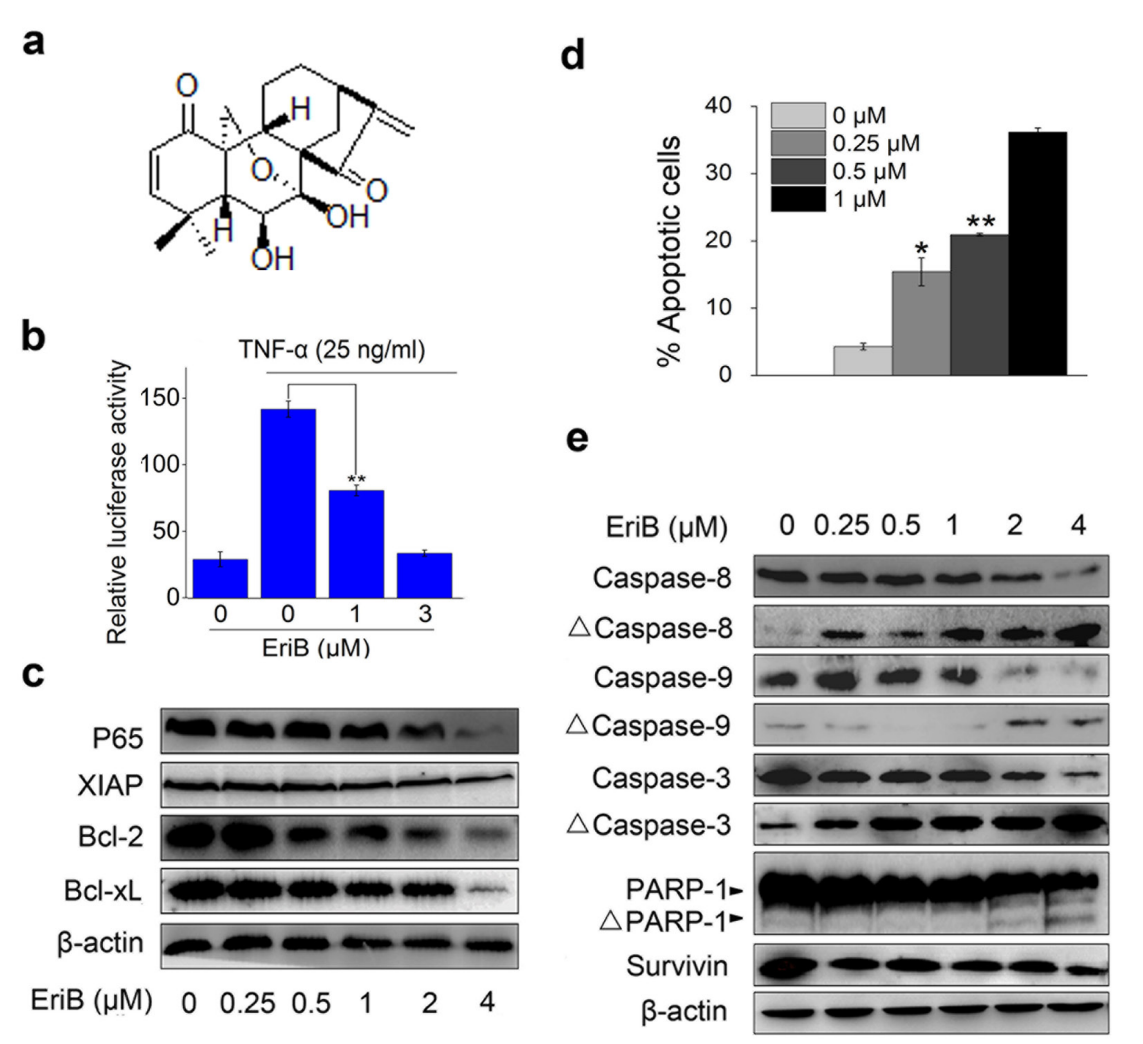
EriB inhibits NF-κB signaling and triggers apoptosis **(A)** Structure of EriB. **(B)** HEK293T cells transiently transfeted with p65-Luc were pretreated with EriB and followed by activation with TNF-α. The reporter activity was determined. The values represent the mean ± S.D. (n=3). **(C)** Western blot analysis of NF-κB downstream target proteins in SMMC-7721 cells with β-actin used as the loading control. **(D)** SMMC-7721 cells were treated with EriB for 48 h. Apoptosis was analyzed by Annexin V-FITC/PI staining. The values represent the mean ± S.D. (n=3). **(E)** Lysates from SMMC-7721 cells treated with EriB for 24 h were subjected to western blot analysis. β-actin was used as the loading control. Statistical significance was analyzed by One-way ANOVA, **p<0.01, *p<0.05.

Isodon species (Labiatae) are widely distributed plants, many of which are used for the treatment of cancer and inflammation in Chinese folk medicine. Over the past twenty years, the structures and bioactivities of their diterpenoids constituents, especially those with an *ent*-kaurane skeleton, have received considerable phytochemical and biological attention [1]. Eriocalyxin B (EriB), an *ent*-kaurene diterpenoid (structure designated in Figure 1a) isolated from *Isodon eriocalyx var. laxiflora*, has been shown potent anti-tumor activity both *in vitro* and *in vivo*, and may be a promising candidate as an antitumor agent. A multitude of studies have been undertaken to elucidate the underlying mechanisms of the anti-tumor activity of EriB. NF-κB, MAPK/ERK, AKT and P53 pathways have been reported, among which the NF-κB signaling is largely involved [2–7]. NF-κB signaling regulates inflammation, tumorigenesis, cancer development, metastasis [8] and chemoresistance [9, 10]. The five members of NF-κB family, known as RelA (p65), RelB, c-Rel, p50/p105 (NF-κB1), and p52/p100 (NF-κB2), are sequestered in the cytoplasm as either homodimers or heterodimers. Upon activation, the inhibitory subunit IκBα is phosphorylated, ubiquitinated and degraded, which promotes the translocation of the NF-κB complex into the nucleus and activates the expression of the target genes [11, 12]. It was proposed that EriB-mediated apoptosis of acute myeloid leukemia cells and the ovarian cancer stem cells was associated with NF-κB inactivation by preventing the IκBα degradation and the subsequent nuclear translocation of NF-κB [2, 3]. Interfering with the binding of NF-κB subunits to the DNA response element without changing the translocation of NF-κB or their dimerization was reported to be responsible for cell apoptosis induced by EriB as well [4, 5]. EriB inhibition of NF-κB pathway also contributed to the attenuation of autoimmune inflammation [6].

Though extensive investigations of the mechanism involved in the inhibition of NF-κB signaling by EriB have been reported, despite of some controversies, the direct targets of EriB remain unknown. To address the problem, we resorted to the technology of activity-based protein profiling (ABPP) strategy for solutions [13]. A series of labeled EriB probes were designed and synthesized by conjugating fluorophore or biotin with EriB. With the labeled activity-based probes, we investigated the direct target of EriB and the detailed mechanisms involved in its anti-tumor activity. Our data showed that EriB covalently modifies cysteine 62 of p50 through the α,β-unsaturated ketones and EriB induces cancer cell apoptosis through interfering with the binding between NF-κB and the response elements by directly targeting the cysteine 62 of p50.

## RESULTS

### EriB inhibits NF-κB signaling and triggers cell apoptosis

To begin with, the effect of EriB on the NF-κB activity was investigated with reporter activity assay. We observed that EriB significantly inhibited TNF-α-induced activity of NF-κB reporter (p65-Luc) in a concentration-dependent manner (Figure 1b). Further, we examined the effect of EriB on the expression of the endogenous target genes of NF-κB signaling. Western blot analysis showed that EriB suppressed the expression of RelA [14] and XIAP [15] in SMMC-7721 cells. The anti-apoptotic proteins Bcl-2 and Bcl-xL, also known the downstream target genes of NF-κB [16], were down-regulated as well by EriB when the concentration is over 0.5 μM (Figure 1c). Taken together, EriB inhibited both intrinsic and TNF-α-induced NF-κB activation.

Then the effect of EriB on cell apoptosis was checked by Annexin V-FITC/PI double staining. The result showed that EriB induced obvious apoptosis in SMMC-7721 cells with a dose-dependent manner (Figure 1d), whereas no evident cell cycle arrest was observed in EriB-treated SMMC-7721 cells (Supplementary Information, Figure S1). There are two main pathways involved in apoptosis: one is the extrinsic pathway related to the activation of caspase-8, and the other is the intrinsic pathway regulated by Bcl-2 family proteins and activation of caspase-9, which converge at caspase-3 and follow by the activation of executive caspases [17, 18] and PARP-1 [19]. With western blotting assay, our results demonstrated that the activation of both caspase-8 and caspase-9, as well as the cleavage of caspase-3 and PARP-1, were increased in EriB treated SMMC-7721 cells, whereas the expression of survivin, an inhibitor of caspase-3, 7 and 9 [20], was down-regulated (Figure 1e). Our findings showed that EriB induces tumor cell apoptosis as is associated with inhibited NF-κB signaling, which is consistent with previous studies [2–6].

### Design and synthesis of activity-based probes of EriB

**Figure 2:**
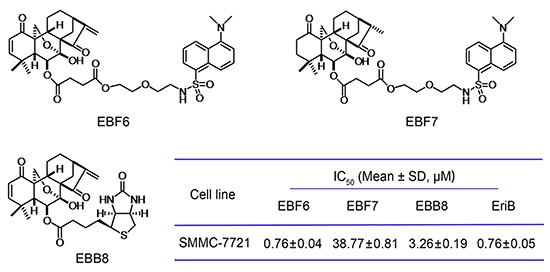
Chemical structures of EBF6, 7, EBB8 and their cytotoxicity against SMMC-7721 cells

Though extensive investigations of the mechanism involved in the inhibition of NF-κB signaling by EriB have been reported, despite of some controversies, the direct targets of EriB remain unknown. To identify the cellular target of EriB and further elucidate the molecular mechanisms underlying the anti-tumor effects of EriB, the activity-based probes (ABPs) were designed and synthesized (Figure 2) (Supplementary Information, Figure S2). Typically, the ABP contains three components: the substrate that binds with the target protein, the hydrophilic spacer and the reporter, which is either a fluorophore for direct and sensitive detection, or a biotin to facilitate the isolation and enrichment of target proteins in complex proteomes. On the basis of the structure-activity relationship (SAR) studies of EriB [21], we know that the substitutions at C-6 hydroxyl group exert minimal implication on the activity. What's more, the C-6 hydroxyl group is highly chemically accessible. So the C-6 hydroxyl group was chosen as the site for the attachment. With polyethylene glycol as the hydrophilic linker, we installed a dansyl tag, the fluorophore commonly used in biological studies [22], to the C6 hydroxyl group to yield dansyl EriB (EBF6). Biotinyl EriB (EBB8) was directly accessed by condensation of biotin with EriB. EBF7, dansyl EriB with reduced α, β-unsaturated ketones, was generated by catalytic hydrogenation of EBF6 with Pd/C under H_2,_ whose stereochemistry was characterized by NOESY. The cytotoxicity of the synthesized probes was determined, with the parent compound EriB used as the control (Figure 2). Both EBF6 and EBB8 exhibited comparable cytotoxicity toward SMMC-7721 cells to that of EriB. As expected, EBF7 lost the cytotoxicity due to the destroyed α, β-unsaturated ketones, which were proved to be the key pharmacophores for the anti-tumor activity of EriB through structure-activity relationship studies [21].

### EriB directly binds to p50

**Figure 3:**
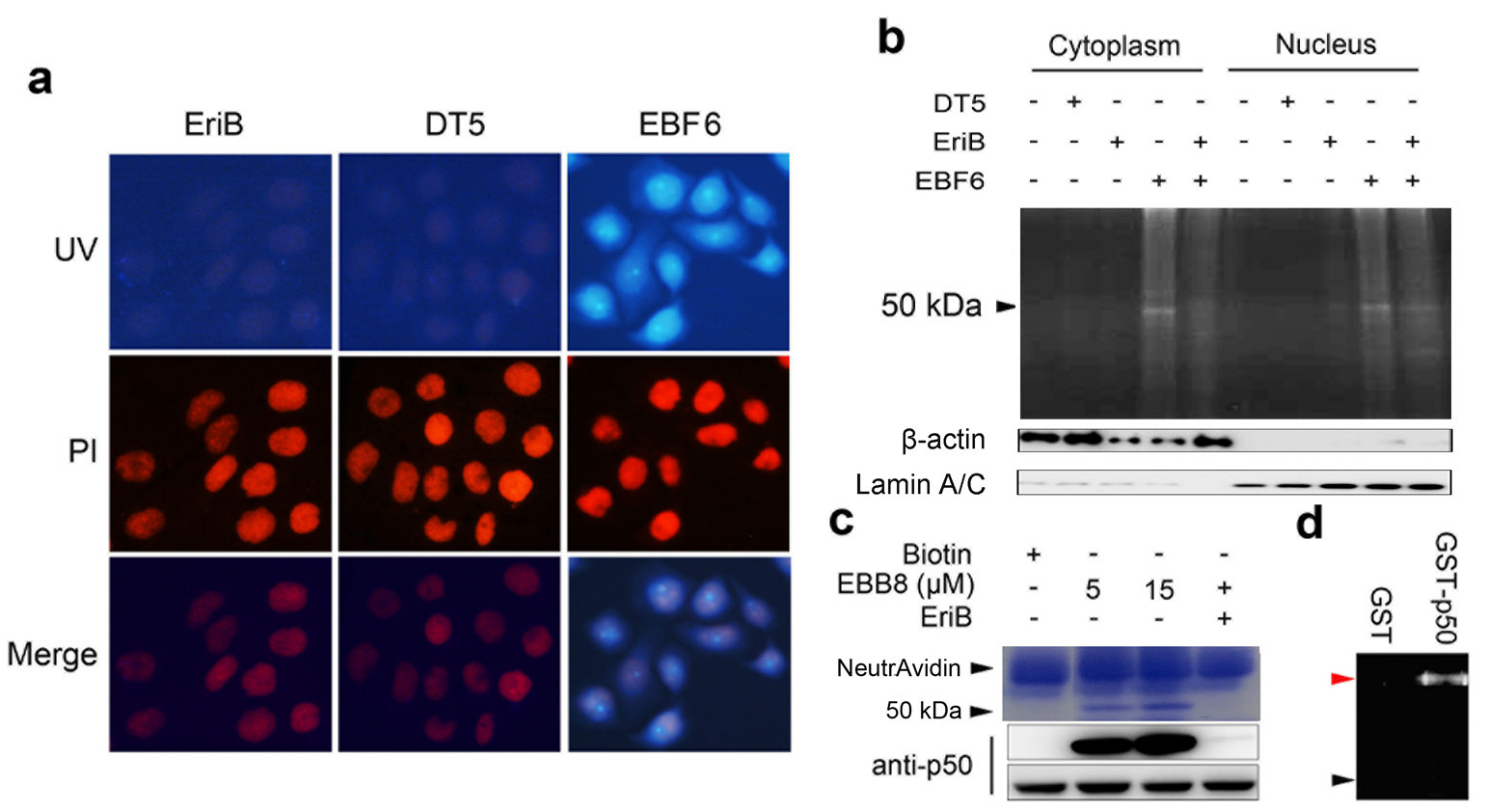
EriB selectively interacts with p50 **(A)** SMMC-7721 cells were incubated with 1 μM EriB, DT5 (dansyl tag alone, **Supporting Information**) and EBF6 for 3 h, respectively, and the fluorescence was observed under microscopy. Nucleus and EBF6 were recognized by the red and blue fluorescence respectively. **(B)** The cytoplasmic fraction and nucleic fraction of SMMC-7721 cell lysates were incubated with EBF6 (1 μM), with or without the presence of EriB, followed by resolving with SDS-PAGE and imaging under UV transilluminator. β-actin and Lamin A/C are the loading controls of cytoplasmic fraction and the nucleic fraction respectively. **(C)** SMMC-7721 cell lysates were incubated with EBB8 with or without the presence of EriB, or with biotin alone, followed by pull-down with streptavidin beads. The precipitates were resolved by SDS-PAGE, and the gel was stained with coomassie brilliant blue or subjected to immuno-blotting with p50 antibody, with p50 as the input. **(D)** Purified human recombinant GST and GST-p50 were incubated with EBF6 (1 μM), respectively, for 3 h and after SDS-PAGE the samples were exposed to UV light. The red arrow indicates the GST-p50, and black arrow denotes GST.

Next we extended our studies to elucidate the cellular target of EriB using the synthetic ABPs. It was observed that EBF6 distributed in both cytoplasm and nucleus of treated SMMC-7721 cells (Figure 3a). To directly visualize the proteins labeled by EriB, cytoplasmic and nucleic lysates of SMMC-7721 cells were fractionated and incubated with EBF6 (1 μM), respectively, followed by SDS-PAGE. The EBF6 bound proteins were detected with fluorescence under a UV trans-illuminator (UVP). As shown in Figure 3b, the cytoplasm and nucleus were successfully separated with the different expression of β-actin and Lamin A/C as the demonstration and a specific protein band with molecular weight of 50 kDa was detected both in cytoplasmic and nucleic fractions (lane 4 and 9), whereas no any band was detected under the treatment of either EriB or dansyl tag alone (DT5, Supplementary Information) (lane 2, 3 and 7, 8). Moreover, the fluorescence of the labeled 50-kDa protein band was competed off in the presence of a 3-fold excess of EriB both in cytoplasm and nucleus (Figure 3b, lane 5 and 10), indicating EriB binds with the 50-kDa protein specifically. When the cell lysates were treated with high concentration of EBF6 (5 μM), a few proteins were labeled and visualized, among which the 50-kDa band appeared to be the predominant one (Supplementary Information, Figure S3).

As one crucial regulator of NF-κB signaling, p50, a protein of 50 kDa, dominantly dimerizes with p65 and is sequestered in the cytoplasm with the inhibitory subunit IκB proteins in resting cells. Activation of NF-κB signaling pathway leads to the release and translocation of p50/p65 dimer to the nucleus, and then activates the transcription of the target genes [23, 24]. So we postulated that the protein weighing 50 kDa labeled by EriB was p50.

To verify our speculation, the biotinylated EriB (EBB8) was generated and incubated with SMMC-7721 cell lysates to directly pull down the potential proteins bound to EriB, with the streptavidin-coated beads as the immobilization strategy. As shown in Figure 3c, a 50-kDa protein was captured by EBB8. When the membrane was subjected to immuno-blotting, the 50-kDa band was recognized specifically by p50 antibody. Moreover, the interaction between EBB8 and p50 could be effectively competed by the co-presence of 10-fold excess of EriB, which was consistent with the results observed in the competition experiment performed with EBF6, the fluorophore probe of EriB. The interaction of EriB with p50 was also observed in HEK293T cells. In HEK293T cells, treatment of TNF-α caused a decrease of p105 and an increase of p50 as reciprocation [25]. At the meanwhile the augment of p50 captured by EBB8 was detected in TNF-α stimulated cells and EriB competed the interaction between EBB8 and p50 (Supplementary Information, Figure S4).

To further confirm the binding between EriB and p50, purified human recombinant GST-p50 was incubated with EBF6, with GST protein as a control. The result showed that EBF6 selectively interacted with p50 rather than the GST protein (Figure 3d).

### EriB interferes with the binding of p50 to the response elements

**Figure 4:**
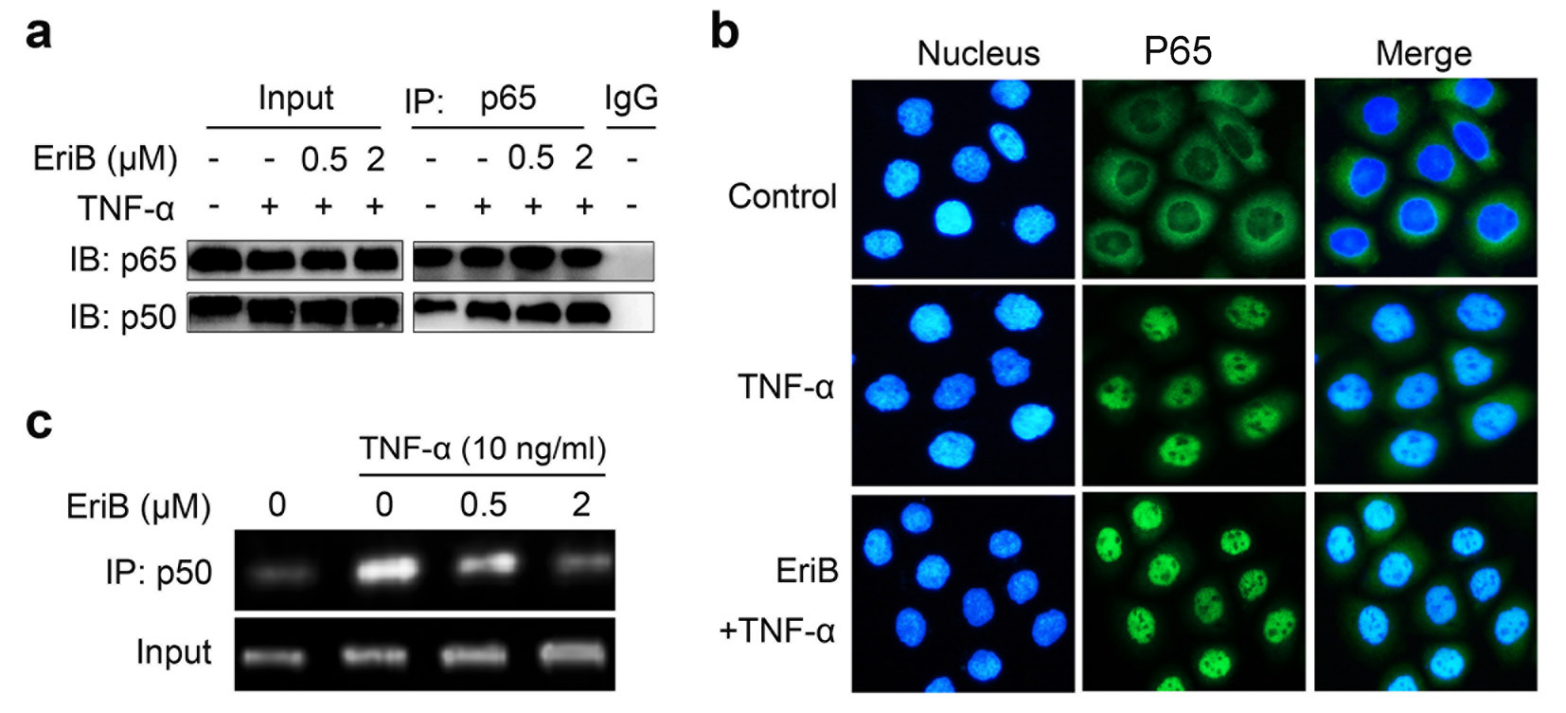
EriB interferes with the binding of p50 to the response elements **(A)** SMMC-7721 cells were preincubated with EriB for 3 h before TNF-α (10 ng/ml) stimulation. Immunoprecipitation was performed with anti-p65 antibody. P65 and the associated p50 were detected by immunoblotting. P65 and p50 were used as the inputs. **(B)** SMMC-7721 cells were pretreated with EriB (0.5 μM) before TNF-α (10 ng/ml) stimulation. Distribution of NF-κB was examined by immunofluorescence staining. NF-κB and nucleus were recognized by the green and blue fluorescence, respectively. **(C)** SMMC-7721 cells were incubated with EriB at the indicated concentrations for 3 h before TNF-α stimulation. Chromatin immunoprecipitation assay was performed using p50 antibody. NF-κB-associated DNA was analyzed by PCR with the histone 3-associated RPL30 DNA as the input.

The hallmark of the activation of NF-κB signaling is the translocation of p50/p65 dimer from the cytoplasm to the nucleus, and then binding to the response elements to activate the transcription. We explored the effects of the binding of EriB with p50 on these events. We first determined whether EriB interfered with the association of p50 and p65 in SMMC-7721 cells. Co-immunoprecipitation assay showed that TNF-α treatment increased the dimerization of p50 with p65, while pretreatment of EriB exerted little effects on the amount of p65-associated p50 (Figure 4a).

Next we examined the nuclear translocation of NF-κB by immunofluorescence staining of p65 protein. As shown in Figure 4b, obvious translocation of NF-κB was observed 30 min after TNF-α treatment. Pre-incubation of the cells with EriB did not block the translocation of NF- κB stimulated by TNF-α, indicating that EriB does not affect the nuclear translocation of NF-κB either.

Subsequently, the effect of EriB on the interaction of NF-κB with the DNA response elements was investigated with chromatin immuno-precipitation assay. The results demonstrated that TNF-α markedly promoted the binding activity of NF-κB with the promoter of Bcl-xL as previously reported [26], whereas, which was effectively blocked by EriB dose-dependently (Figure 4c). Taken together, EriB, through direct interacting with p50, interfered with the DNA binding activity of p50 without affecting the dimerization or translocation of p50/p65.

### EriB covalently modifies Cys62 of p50 through the α, β-unsaturated ketones

**Figure 5:**
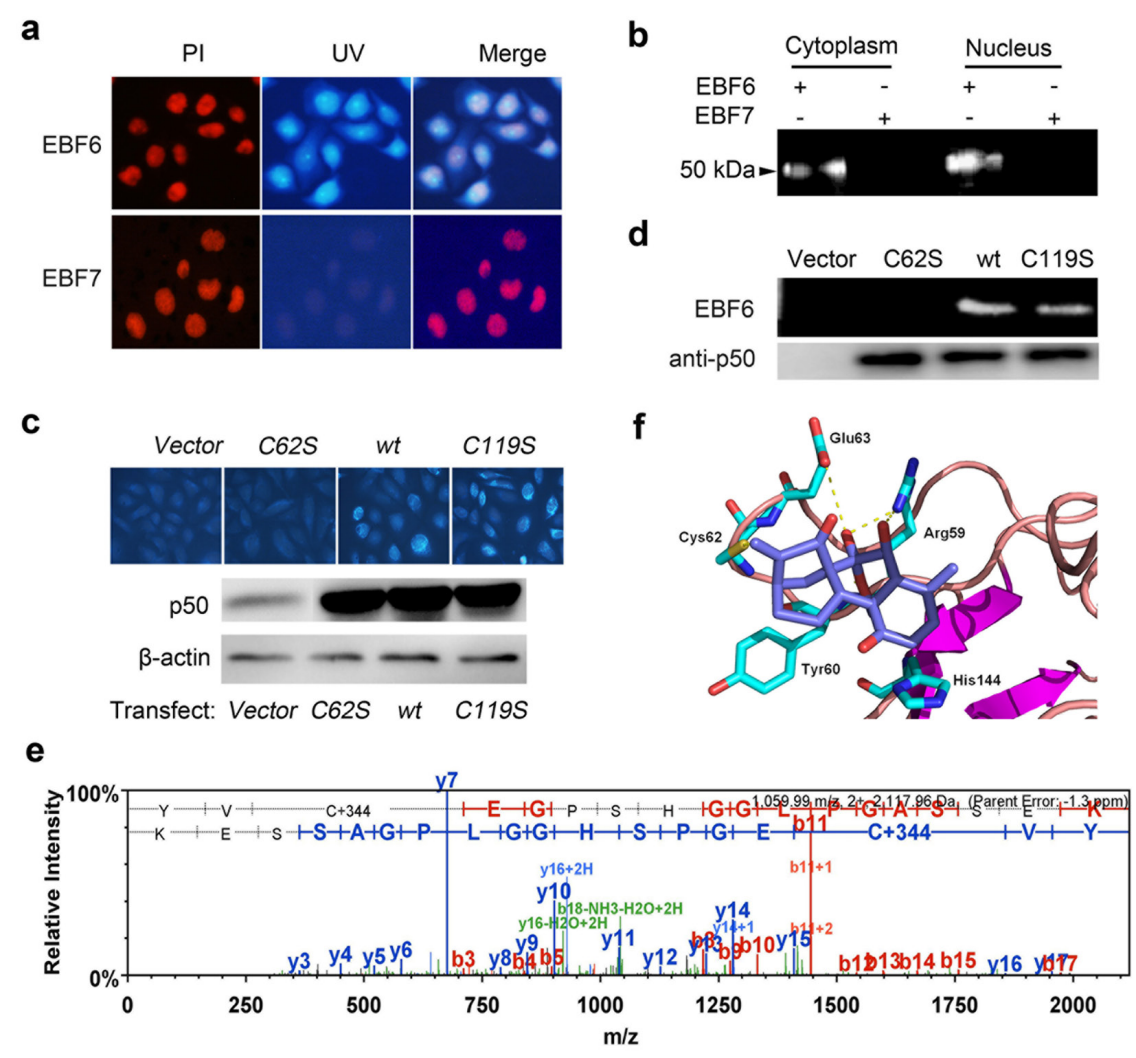
EriB directly targets the cysteine 62 of p50 through α, β-unsaturated ketones **(A)** SMMC-7721 cells were incubated with 1 μM EBF7 (probe of reductive EriB) or EBF6, and the fluorescence was observed under microscopy. Nucleus and EBF6 were recognized by the red and blue fluorescence respectively. **(B)** Cytoplamic and nucleic fractions of SMMC-7721 cells lysates were treated with 1 μM EBF7 or EBF6. After SDS-PAGE, the samples were exposed to UV light. **(C)** SMMC-7721 cells were transfected with *p50^WT^*, *p50^C62S^* and *p50^C119S^*, respectively, and treated with EBF6 (3 μM) for 3 h. The fluorescence was detected under microscope. The p50 antibody was used to detect the expression of the protein with Western blot assay. **(D)** The purified FLAG tagged human p50 and p50 variant proteins were incubated with 1 μM EBF6 for 1 h respectively. After SDS-PAGE, the samples were exposed to UV light with p50 as the loading control. **(E)** Human recombinant p50 protein was incubated without or with EriB (1 μM) and subjected to liquid chromatography-mass spectra analysis. **(F)** The binding model of EriB in the DNA binding region (DBR) of p50. The yellow dotted lines represent hydrogen bonds between EriB (cyan) and the related residues of p50 (blue).

Structure-activity relationship studies of EriB revealed that the α, β-unsaturated ketones were the key pharmacophores for the anti-tumor activity of EriB as previous reported [21]. To elucidate the structural basis of the binding of EriB with p50, in our present study, EBF7, dansyl EriB with α, β-unsaturated ketones reduced was created, which, as expected, lost the anti-tumor activity *in vitro* (IC_50_>35 μM in MTT assay) (Figure 2). Investigations were performed to verify the binding between p50 and EBF7. As expected, no fluorescence was observed in SMMC-7721 cells upon the treatment of EBF7 (Figure 5a). Consistently, the 50-kDa protein band on the gel was abolished as well from both the EBF7 treated cytoplasmic and the nuclear fractions (Figure 5b). These results indicated that the loss of binding capacity of EriB resulted from the reduction of α, β-unsaturated ketones and thus α, β-unsaturated ketones are crucial motifs for EriB to interact with p50.

α, β-unsaturated ketones are known as Michael acceptors that potentially capture well-positioned nucleophiles at its target binding site, and may react with sulfhydryl groups of cysteine residues of proteins [27, 28]. Human p50 has seven cysteine residues at positions 62, 88, 119, 124, 162, 262, and 273 [29, 30]. It is known that residues 59-71 (RYVCEGPSHGGLP) of p50 constitute the DNA binding domain of NF-κB [31–34], among which the sulphydryl group of Cys62 is an important determinant of DNA recognition by p50 subunit of NF-κB [35]. To investigate if Cys62 of p50 is the residue that EriB bound with, the mutants of p50 62Cys→Ser (*p50^C62S^*) and p50 119Cys→Ser (*p50^C119S^*) were constructed. SMMC-7721 cells transfected with wild type p50 (*p50^wt^*), *p50^C62S^* and *p50^C62S^* plasmids were treated with EBF6 respectively, and were observed under the fluorescence microscope. Compared with the vector transfected group, the intensities of the fluorescence obviously increased in both *p50^wt^* and *p50^C119S^* transfected cells, while hardly changed in the *p50^C62S^* transfected cells (Figure 5c), suggesting the Cys62 is required for the binding of EriB with p50.

FLAG tagged proteins of p50wt and the mutant p50C62S and p50C119S were expressed in SMMC- 7721 cells and purified, and then incubated with EBF6 respectively, followed by SDS-PAGE (Figure 5d). Compared with the wide type p50 and p50C119S, the interaction between EBF6 and p50C62S was abolished, which confirmed EriB bound to the cysteine 62 of p50. The covalent modification of cysteine 62 of p50 by EriB was further confirmed by mass spectra analysis. As shown in Figure 5e, a 344 Da increase (the molecular weight of EriB) was found at the Cys62 of the peptide (YVCEGPSHGGLPGASSEK) of p50 (Figure 5e).

Besides, the binding model of EriB to p50 was demonstrated based on molecular docking. As shown in Figure 5f, the modeled EriB-p50 complex has the Michael acceptor at C-17 which could form a C-S bond with Cys62, and the following favorable interaction between EriB and p50 were also observed: a hydrogen bond between the C-7 hydroxyl group with the Glu63 carbonyl group and the hydrogen bonds between the C-6, C-7 hydroxyl groups and the Arg59 NH group.

### P50 knock-down attenuates the cytotoxicity and the apoptosis induced by EriB

**Figure 6:**
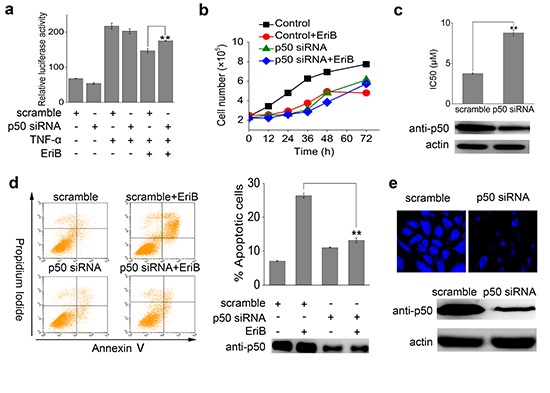
P50 knock-down attenuates the cytotoxicity and apoptosis inducing activity of EriB **(A)** HEK 293T cells were transiently transfected with scrambled siRNA or p50 siRNA for 48 h, then pretreated with 1 μM EriB and stimulated with 25 ng/mL TNF-α for 18 h. Cells were subjected to the analysis of NF-κB luciferase activity. The values represent the mean ± S.D. (n=3). **(B)** The SMMC-7721 cells were transfected with p50 small interfering RNA for 48 h, and the cell count was documented at 0, 12, 24, 36, 48 and 72 h in the absence or presence of EriB (1 μM), respectively. The values represent the mean ± S.D. (n=3). **(C)** Cytotoxic effects induced by EriB after 48 h treatment toward SMMC-7721 cells transfected with p50 siRNA were determined using MTT assays. The values represent the mean ± S.D. (n=3). **(D)** SMMC-7721 cells were treated with EriB (1 μM) for 48 h after p50 siRNA transfection. Apoptosis was analyzed by Annexin V-FITC/PI staining and the ratios of apoptotic cells were quantified. The corresponding p50 level was analyzed by Western blotting. The values represent the mean ± S.D. (n=3). **(E)** SMMC-7721 cells were transfected with p50 siRNA and treated with EBF6 (3 μM) for 3 h. The fluorescence was detected under microscope. The corresponding p50 level was analyzed by Western blotting. All the experiments were repeated three times. The values represent the mean ± S.D. (n=3). Statistical significance was analyzed by One-way ANOVA, **p<0.01.

To investigate whether p50 is required for EriB to exert its anti-tumor activity, cell proliferation was studied in cells with p50 knocked down by small interfering RNA (siRNA). Luciferase assay showed that p50 knock-down compromised the NF-κB transcriptional activity suppressed by EriB (Figure 6a). According to the growth curve analysis (Figure 6b), EriB showed obvious inhibitory effects on the proliferation of SMMC-7721 cells (difference between the red curve and the black line). As a result, siRNA mediated knock-down of p50 in SMMC-7721 cells eliminated the antiproliferative activity of EriB, deduced from the cell growth curves (the green curve and the blue curve in Figure 6b). The IC_50_ of EriB toward cells transfected with p50 siRNA was determined using MTT assay (Figure 6c), with scrambled siRNA as the control. As the result showed, transfection of p50 siRNA attenuated efficiently the cytotoxic activity of EriB, though not remarkable due to probably the relatively low efficiency of interference. Cell apoptosis assay revealed that the apoptotic effect of EriB was markedly weakened in SMMC-7721 cells with p50 knocked down by siRNA (Figure 6d). Furthermore, the intensity of the fluorescence of bound EriB to p50 decreased obviously in p50 knocked-down SMMC-7721 cells, comparing with the control cells (Figure 6e). These data suggest that the interaction between EriB and p50 is required for the anti-tumor activity of EriB.

## DISCUSSION

As an ent-kaurene diterpenoid isolated from Isodon eriocalyx var. Laxiflora, Eriocalyxin B (EriB) possesses potent bioactivity of antitumor and anti-autoimmune inflammation and has received largely the attention from both chemistry and biology. Though extensive investigations of the mechanism involved in the inhibition of NF-κB signaling by EriB have been reported, despite of some controversies, the direct targets of EriB remain elucidated, which became the aim of this resent study. Our findings showed that EriB-induced tumor cell apoptosis was associated with inactivation of NF-κB signaling, which is consistent with previous studies [2–6]. We then synthesized the activity-based probes (ABPs) of EriB and with the favor of the ABBP technology, we identified p50 protein as the direct cellular target of EriB in SMMC-7721 cells.

P50 is one of the key regulators of NF-κB signaling, which forms either homodimers or heterodimers and translocates into nucleus and activate the transcription of the target genes of the pathway upon the activation of the signaling. Either the dimerization or the cellular distribution of p50/p65 in our current study turned out to be not affected by the binding of EriB with p50, which is consistent with previous studies [4, 5]. It was also reported that EriB-mediated NF-κB inactivation of acute myeloid leukemia cells and the ovarian cancer stem cells was associated with the inhibition of nuclear translocation of NF-κB [2, 3]. We assumed that the difference could be due to the different types of cells tested and experimental conditions, such as the doses and time used for EriB treatment.

The direct interaction between EriB and Cys62 of p50 was confirmed in the present study by mass spectra analysis which showed that EriB directly interacted with the Cys62 of p50. Based on the kinetic assay with total nuclear lysates, it was showed that EriB works non-competitively to inhibit NF-κB /DNA binding in previous studies [4]. Our data may suggest EriB inhibits the binding of p65/p50 complex to DNA through direct interaction with p50.

At the last part, the present study further showed that the interaction between EriB and p50 is crucial for the antitumor activity of EriB, which verified that functionally p50 is the cellular antitumor target of EriB. Down-regulation of p50 using small interfering RNA attenuated the NF-κB transcriptional activity, antiproliferative activity, cytotoxic activity and apoptotic activity induced by EriB in SMMC-7721 cells, which might be due to the possibility that other NF-κB family members, which EriB cannot interfere with, participate the signaling transduction and activate the NF-κB pathway [36].

In our present study, a couple of proteins appeared relatively weakly on the gel when pulled down with the dansyl EriB besides p50. Though we couldn't ruled out the possibility that EriB may also covalently bind with other proteins at the moment (Supplementary Information, Figure S3), we propose p50 is the major antitumor target of EriB based on our findings.

## CONCLUSION

Activity-based protein profiling is a forward-chemical proteomic tool that uses specially designed chemical probes to fish mechanistically-related protein [13, 37]. With the favor of the technology, we identified p50 protein as the direct target of EriB for the first time and illustrated the structural basis for their binding. The fact that EriB has already proved to be effective suppressing tumor growth both *in vitro* and *in vivo* highlights that p50 may be a very promising drug target for cancer therapy and EriB can be used as a natural chemical template for the development of p50 targeted therapeutic agents.

## MATERIALS AND METHODS

### Isolation of EriB and synthesis of the ABPs

Synthetic schemes, experimental procedures and characterization data of all new compounds are detailed in Supplementary Information.

### Cells and plasmids

Hepatocellular carcinoma SMMC-7721 and HEK293T cells were obtained from Shanghai cell bank in China. All the cells were cultured in RPMI-1640 or DMEM medium (HyClone, Logan, UT), supplemented with 10% fetal bovine serum (HyClone, Logan, UT), 100 units/mL penicillin and 0.1 mg/mL streptomycin (HyClone) at 37 °C in a humidified atmosphere with 5% CO_2_. *p50^wt^*, *p50^C62S^* and *p50^C119S^* were constructed by site-directed mutagenesis based on pCMV-p105 (Thermo Scientific, Rockford, IL).

### MTT assay

Cytotoxicity of compounds was determined by MTT method. Briefly, cells were plated in 96-well plates 12 h before treatment and continuously exposed to test compounds for 48 h. Then MTT (Sigma-Aldrich, St. Louis, MO) was added to each well. The samples were incubated at 37 °C for 4 h and formazan produced was dissolved by the addition of 200 μL/well of Trevogen, followed by a further incubation at 37 °C overnight in the dark. The optical density (OD) was measured at 595 nm using a microplate reader (Bio-Rad Laboratories). The IC_50_ values are calculated from appropriate dose-response curves.

### Cell apoptosis analysis

Cell apoptosis was analyzed using the Annexin V-FITC/PI Apoptosis kit (BD Biosciences, Franklin Lakes, NJ) according to the manufacturer's protocols. Cells were seeded in 6-well plates at a density of 1.2×10^6^ cells/well. After indicated treatment cells were collected and then washed twice with cold PBS, and then resuspended in a binding buffer containing Annexin V-FITC and propidium iodine (PI). After incubation for 15 minutes at room temperature in the dark, the fluorescent intensity was measured using a FACSCalibur flow cytometer (BD Biosciences, Franklin Lakes, NJ).

### Western blotting

Cells were harvested and lysed in SDS sample buffer (62.5 mM Tris-HCl, pH 6.8, 10% glycerol, 2% SDS, 50 mM DTT and 0.01% bromphenol blue). Lysates were subjected to SDS-PAGE and transferred to PVDF membranes (Millipore). Membranes were blocked with 5% nonfat milk in Tris-buffered saline/0.1% Tween-20 and incubated at 4 °C overnight with the following antibodies: anti-bcl-2 (Santa Cruz, CA), anti-bcl-xL (Santa Cruz, CA), anti-p65 (Santa Cruz, CA), anti-XIAP (Epitomics), anti-survivin (Santa Cruz, CA), anti-caspase 3 (Santa Cruz, CA), anti-cleaved caspase 3 (Santa Cruz, CA), anti-caspase 9 (Epitomics), anti-caspase 8 (Santa Cruz, CA), anti-PARP-1 (Santa Cruz, CA), anti-Lamin A/C (Epitomics) and against β-actin (Santa Cruz, CA), followed by the corresponding horseradish peroxidase-conjugated secondary antibodies. Proteins of interest were visualized and imaged under chemi-luminescent detection using LASmini 4000 (GE Healthcare).

### Cell transfection and luciferase reporter assay

HEK293T cells were transiently transfected with p65-Luc (Beyotime Institute for Biotechnology, China) and pRL-TK (Promega, Madison, WI) plasmids using Lipofectamine 2000 (Invitrogen, Carlsbad, CA) for 3 h in a 48-well plate. Cells were incubated with compounds for 1 h and subsequently activated with TNF-α (Peprotech, Rocky Hill, NJ). Luciferase activities were measured using the Dual-Luciferase Reporter Assay kit (Promega, Madison, WI).

### Nuclear and cytoplasmlic fractionation

Collected cells were lysed in lysis buffer A (10 mM HEPES, 10 mM KCl, 1.5 mM MgCl_2_, 0.5 mM DTT, pH 7.9) with 0.4% Nonidet P-40 and EDTA-free protease inhibitor cocktail (Roche, Indianapolis, IN, USA) for 10 minutes on ice. After being microcentrifuged for 5 minutes at 500 g, the supernatants were collected as cytoplasmic extracts. Pellets were resuspended in lysis buffer B (20 mM HEPES, pH 7.9, 420 mM NaCl, 0.5 mM DTT, 0.2 mM EDTA, and 25% glycerol), and incubated for 30 minutes on ice. After being centrifuged at 12,000 g for 10 minutes, supernatants were collected.

### Pull-down assay

Total cell extracts were lysed and precipitated with biotinyl-EriB for 2 h at room temperature, followed by addition of ImmunoPure immobilized streptavidin beads (Thermo Scientific, Rockford, IL) at 4 °C overnight. After washing for five times with the lysis buffer, the beads were boiled in the presence of the SDS sample buffer. The proteins were separated on 10% SDS-polyacrylamide gels and the specific binding proteins were detected with coomassie brilliant blue or p50 antibody (Epitomics, Burlingame, California).

### Co-immunoprecipitation assay

Extracts of SMMC-7721 cells were incubated with anti-p65 antibody (Santa Cruz, CA) for 2 h, and then incubated with protein G plus/protein A agarose (Santa Cruz, CA) at 4 °C overnight. Samples were washed five times with 50 mM Tris-HCl, pH 7.9, 150 mM NaCl, 1% NP-40 and subjected to Western blotting analysis.

### Immunofluorescence staining

Cells were grown on chamber slides for 18 h before treatment. Drug-treated cells were fixed in 4% paraformaldehyde for 20 minutes. After being washed in PBS, the slides were treated by 0.1 % Triton-X, blocked with 3% BSA in PBS, incubated with anti-p65 antibody (Santa Cruz, CA) overnight, washed with PBS, and incubated with corresponding FITC conjugated secondary antibody (Sigma-Aldrich) for 1 h. The slides were then incubated with DAPI (4′,6-diamidino-2-phenylindole) for 10 minutes and observed under microscopy (Eclipse Ti, Nikon).

### Chromatin immunoprecipitation assay

ChIP was performed in SMMC-7721 cells using SimpleChIP^®^ Enzymatic Chromatin IP Kit (Cell Signaling Technology, Trask Lane Danvers, MA, USA) according to the manufacturer's instructions. The purified DNA was subjected to PCR amplification using the human Bcl-xL promoter specific primers [38]. forward 5′-GCACCACCTACATTCAAATCC-3′ and reverse 5′-CGATGGAGGAGGAAGCAAGC-3′. PCR fragments were analyzed on agarose gel and the size (251 bp) was compared to a molecular weight marker.

### Expression of p50^wt^, p50^C62S^ and p50^C119S^ proteins

SMMC-7721 cells were transfected with *FLAG-p50* and *C62S*, *C119S* mutants for 24 h, and the proteins were purified by ANTI-FLAG^®^ M2 Affinity Gel (Sigma-Aldrich) according to the manufacturer's instructions. The purified proteins were eluted with 3×FLAG peptide (Sigma-Aldrich) and stored at −20 °C for use.

### Liquid chromatography-mass spectra analysis

The detailed protocols were described in Supporting Information.

### Molecular modeling of EriB with p50

EriB was prepared (generating stereoisomers and valid single 3D conformers) by means of the Ligand Preparation module in Discovery Studio 3.5 (Accelrys). The crystal structure of p50 was retrieved from the Protein Data Bank (PDB entry: 3GUT). All crystallographic water molecules and DNA were removed from the coordinate set. Gold 4.0 (CCDC) was used for docking. The DNA binding domain, amino acids 59–71, was defined as the binding pocket on the basis of the previous studies[31–34]. In the docking process, the standard docking score was used to rank the docking conformations. All the parameters were set as the default values, except that a maximum number of 300,000 operations and a population of 200 individuals were imposed. The GoldScore fitness function and ChemScore fitness function were used sequentially, and the best-fitting binding mode was identified.

### Small interfering RNAs

Duplex siRNAs with two thymidine residues (dTdT) at the 3′-end of sequence were synthesized at GenePharma (Shanghai, China). The target sequences were as follows [38]:

p50: 5′-GUCACUCUAACGUAUGCAA-3′.

Scrambled control: 5′-UUCUCCGAACGUGUCA CGU-3′.

SiRNAs were transfected into SMMC-7721 cells using Lipofectamine 2000.

## SUPPLEMENTARY MATERIALS


