# Supplementary material for: Identification and validation of p50 as the cellular target of eriocalyxin B
Source: Oncotarget. 2014 Oct 24;5(22):11354–64. doi: 10.18632/oncotarget.2461 (PMC4294364; doi:10.18632/oncotarget.2461)
Supplement: Supplementary file 1 [file oncotarget-05-11354-s001.pdf]

SUPPLEMENTARY MATERIALS

Cell cycle arrest of EriB in SMMC-7721 cells

Cells were treated with EriB at 0.5, 1, 2 and 4 µM for 24 h. Cells were harvested and washed with PBS and were fixed with 70% ethanol. Then the cells were washed with PBS and incubated with RNase A (Sigma-Aldrich) and stained with propidium iodide (Sigma-Aldrich) and were subjected to cell cycle analysis by FACSCalibur flow cytometry (BD Biosciences, Franklin Lakes, NJ). The distribution of cells of different phases was analyzed by CellQuest Pro (Figure S1).

Isolation of Eriocalyxin B and synthesis of the ABPs

Eriocalyxin B was isolated from leaves of *isodon erioclyx var. laxiflora* according to the reported method [1].

General information for chemistry: All reactions were performed under argon atmosphere using flame-dried glassware unless otherwise noted. CH_2Cl_2 and Et_3N were distilled over CaH_2. THF was distilled over sodium/benzophenone ketyl. All reagents were commercially available and used without further purification unless indicated otherwise. Thin layer chromatographies were carried out on Qing-Dao silica plates (0.25 mm layer thickness). Flash chromatography was performed with 300–400 mesh silica gels. Yields reported were for isolated, spectroscopically pure compounds. ^1H and ^{13}C NMR experiments were performed on a Bruker AM-400 and DRX-500 NMR spectrometer at ambient temperature. The residual solvent protons (^1H) or the solvent carbons (^{13}C) were used as internal standards. ^1H-NMR data are presented as follows: chemical shift in ppm downfield from tetramethylsilane (multiplicity, coupling constant, integration). The following abbreviations are used in reporting NMR data: s, singlet; d, doublet; t, triplet; dd, doublet of doublets; dt, doublet of triplets; m, multiplet. EIMS and HREIMS were taken on a VG Auto Spec-3000 or on a Finnigan MAT 90 instrument. The synthetic schemes are shown as Figure S2.

Preparation of eriocalyxin-6-yl-homo succinic ester (**2**): To a solution of compound **1** (69 mg) and DMAP (10 mg) in DCM (1.5 mL) at 0°C was added Et_3N (101 mg) and succine anhydride (30 mg) successively. The resulting mixture was slowly warmed to the room temperature and stirred for 15 hours until no starting material was detected. A HCl aqueous solution (1M) was added to quench the reaction. The aqueous phase was extracted with EtOAc (15 mL×3). The combined organic layers were washed with H_2O and brine successively,

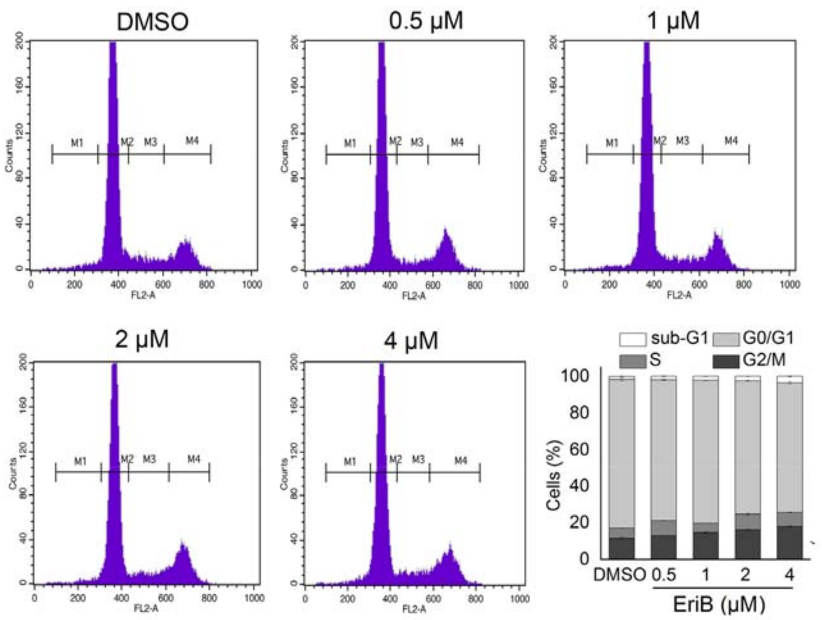

Supplementary Figure S1: EriB induces hardly cell cycle arrest in SMMC-7721 cells. Experiments were done independently in triplicate, and results are reported as means and standard deviations.

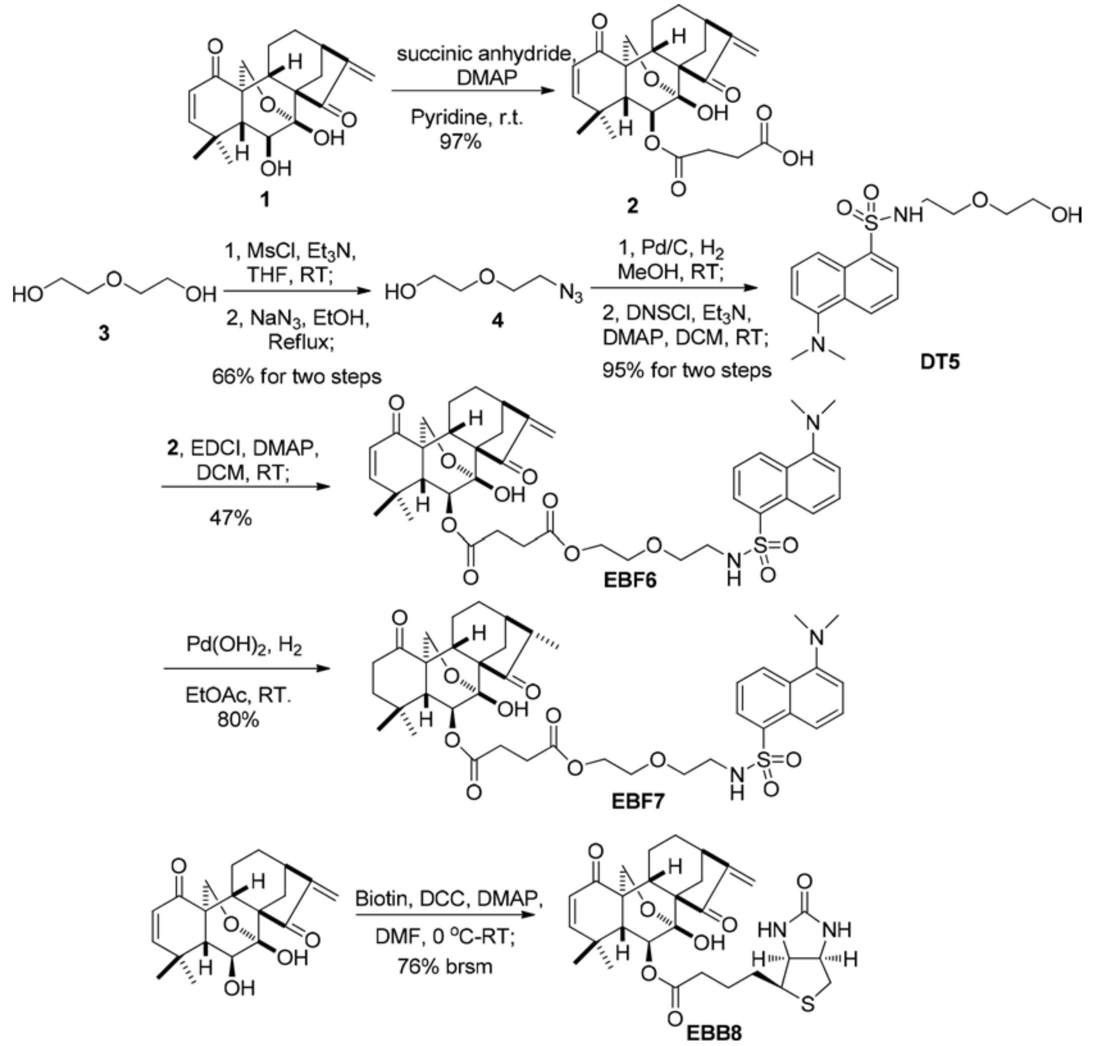

Supplementary Figure S2: Synthetic schemes of EBF 6, EBF7, EBB8.

dried over anhydrous Na_2SO_4 and concentrated. The crude product was subjected to flash chromatography on silica gel (ethyl acetate/petrol ether = 1: 1.5) to give compound **2** (86 mg, 97%) as white solid. ^{1}H NMR (400 MHz, $CDCl_3$) δ 6.84 (d, J = 10.1 Hz, 1H), 5.83 (d, J = 10.1 Hz, 1H), 5.72 (s, 1H), 5.37 (d, J = 8.7 Hz, 1H), 5.29 (s, 1H), 4.94 (s, 1H), 2.99 (m, 1H), 4.27 (d, J = 10.1 Hz, 1H), 3.98 (d, J = 10.1 Hz, 1H), 1. 16 (s, 3H), 1.24 (s, 3H), 2.74 (s, 4H); ^{13}C NMR (100 MHz, $CDCl_3$) δ 202.2 (C), 196.8 (C), 174.7 (C), 173.8 (C), 160.6 (CH), 154.9 (C), 128.0 (CH), 114.6 (CH_2), 97.2 (C), 74.2 (CH), 65.7 (CH_2), 58.6 (C), 53.8 (CH), 48.2 (CH), 47.2 (C), 36.3 (C), 34.3 (CH), 30.5 (CH_2), 30.4 (CH_2), 30.0 (CH_3), 28.9 (CH_2), 26.5 (CH_2), 24.8 (CH_3), 19.6 (CH_2); ESI-MS (*m/z*) 467 $[M+Na]^+$. The spectra data of compound **2** is identical to that described in the literature [2].

Preparation of 2-(2-azidoethoxy) ethanol (**4**): To a solution of polyethylene glycol (16.52 g) in THF (100 mL) was added Et_3N (15 mL) dropwise. The resulting mixture was stirred at room temperature for 0.5 hour. Then the mixture was immersed in water-ice bath. And MsCl was added to the mixture dropwise. The mixture was slowly warmed to the room temperature, and stirred overnight. Then the solvent was evaporated off. EtOH (100 mL) and NaN_3 (6.5 g) was added successively. The mixture was heated to reflux for 24 hours. Then the solvent was evaporated off, and saturated NaCl aqueous solution was added to dilute the mixture. The aqueous phase was extracted with EtOAc (200 mL×3). The combined organic layers were washed with brine, dried over anhydrous Na_2SO_4 and concentrated. The crude product was subjected to flash chromatography on silica gel (ethyl

acetate/petrol ether = 1: 3) to give compound **4** (17.02 g, 85%) as colorless oil. The structure of compound **4** was assigned by comparison the spectra data with that of the literature [3].

Preparation of 5-(dimethylamino)-N-(2-(2-hydroxyethoxy)ethyl)naphthalene-1- sulfonamide (**DT5**): step 1: To a solution of compound **4** (143 mg) and in MeOH (20 mL) under H_2 was added Pd/C (14 mg) in one portion. The mixture was stirred at room temperature for half an hour until no starting material was detected. The mixture was filtered through celite. The filtrate was concentrated under vacuum. The crude product was used for next step without purification.

Step 2: To a solution of the crude product (93 mg) and DMAP (5 mg) in THF (5 mL) was added Et_3N (122 mg) dropwise. The resulting mixture was stirred at the room temperature for 0.5 hour. Then DNSCl (106 mg) was added to the mixture at 0 °C in two portions. The resulting mixture was further stirred for 4 hours until no starting material was detected by TLC. HCl aqueous solution (1 M) was added to quench the reaction. The aqueous phase was extracted with EtOAc (20 mL×3). The combined organic layers were washed with H_2O and brine successively, dried over anhydrous Na_2SO_4 and concentrated. The crude product was subjected to flash chromatography on silica gel (ethyl acetate/petrol ether = 1: 1.5) to give compound **DT5** (99 mg, 71%) as green yellow film. The structure of **DT5** was assigned by comparison the spectra data with that of the literature [4].

Preparation of 6-O-[5-(dimethylamino)-naphthalene-1-sulfonamide-N-2-ethyl-1- (1-ethoxyl)-2-hydroxyl]-succinic ester-yl] Eriocalyxin B (**EBF6**): To a solution of compound **2** (11 mg) and **DT5** (7 mg) in DCM (1.0 mL) at 0 °C was added EDCI (8 mg) and DMAP (5 mg) successively. The resulting mixture was slowly warmed to the room temperature and stirred for 5 hours until no starting material was detected. H_2O was added to quench the reaction. The aqueous phase was extracted with EtOAc (10 mL×3). The combined organic layers were washed with brine, dried over anhydrous Na_2SO_4 and concentrated. The crude product was subjected to flash chromatography on silica gel (Chloroform : MeOH = 30 : 1) to give **EBF6** (17 mg, 85%) as green yellow foam. ^{1}H NMR (400 MHz, $CDCl_3$) δ 8.54 (d, J = 8 Hz, 1H), 8.32 (d, J = 8 Hz, 1H), 8.24 (dd, J = 8 Hz, 4Hz, 1H), 7.57 (t, J = 8 Hz, 8Hz, 1H), 7.51 (t, J = 8 Hz, 8Hz, 1H), 7.19 (d, J = 8 Hz, 1H), 6.74 (d, J = 12 Hz, 1H), 5.85 (d, J = 8 Hz, 1H), 5.59 (t, J = 8 Hz, 4Hz, 1H), 5.37 (d, J = 8 Hz, 1H), 5.28 (s, 1H), 4.44 (s, 1H), 4.36 (d, J = 12 Hz, 1H), 4.10-4.14 (m, 1H), 4.00-4.04 (m, 2H), 3.31-3.37 (m, 4H), 3.14 (dd, J = 12 Hz, 4Hz, 2H), 2.99 (dd, J = 12 Hz, 4Hz, 1H), 2.88 (s, 6H), 2.87 (d, J = 8 Hz, 2H), 2.66-2.72 (m, 1H), 2.49 (d, J = 8 Hz, 1H), 2.29 (d, J = 12 Hz, 1H), 2.12 (d, J = 8 Hz, 1H), 2.07 (d, J = 12 Hz, 1H), 1.98 (dd, J = 8 Hz, 4Hz, 1H), 1.66 (s, 2H), 1.42 (d, J = 8 Hz, 2H), 1.40 (s, 2H), 1.37 (d, J = 8 Hz, 1H), 1.31 (d, J = 8 Hz, 1H), 1.25 (d, J = 8 Hz, 1H), 1.23 (s, 3H), 1.17 (s, 3H), 0.91 (t, J = 8 Hz, 8Hz, 1H), 0.89 (d, J = 8 Hz, 1H); ^{13}C NMR (100 MHz, $CDCl_3$) δ 202.4 (C), 196.5 (C), 175.1 (C), 172.1 (C), 160.1 (CH), 153.0 (C), 151.8 (C), 135.1 (C), 130.8 (CH), 130.3 (CH), 129.8 (C), 129.6 (C), 128.7 (CH), 128.3 (CH), 127.4 (CH), 123.1 (CH), 118.8 (CH), 115.6 (CH_2), 115.1 (CH), 96.6 (C), 74.1 (CH), 69.2 (CH_2), 68.7 (CH_2), 68.1 (C), 65.3 (CH_2), 63.2 (CH_2), 57.6 (C), 52.7 (CH_2), 47.1 (CH_2), 46.5 (C), 45.3 (CH_3), 42.8 (CH_2), 35.7 (C), 33.4 (CH), 29.6 (CH_2), 29.3 (CH_2), 29.2 (CH_3), 28.8 (CH_2), 25.6 (CH_2), 24.7 (CH_3), 18.8 (CH_2); HR-ESI-MS (*m/z*): 765.3044 $[M+H]^+$ (calcd. for $C_{40}H_{49}N_2O_{11}S$, 765.3057).

Preparation of 6-O-[5-(dimethylamino)-naphthalene-1-sulfonamide-N-2-ethyl-1- (1-ethoxyl)-2-hydroxyl]-succinic ester-yl] 2,3,16,17-tetrahydro Eriocalyxin B (**EBF7**): To a solution of **EBF6** in EtOAc (1 mL) was added Pd/C (1 mg) in one portion. The mixture was stirred at room temperature under H_2 for 3 hours until no starting material was detected by TLC. Then Pd/C was filtered off. The filtrate was evaporated under vacuo. The crude product was subjected to preparative TLC to afford **EBF7** (2.9 mg) green yellow foam. ^{1}H NMR (600 MHz, $CDCl_3$) δ 8.47 (d, J = 8.5 Hz, 1H), 8.23 (d, J = 8.6 Hz, 1H), 8.18 (d, J = 7.2 Hz, 1H), 7.51 (t, J = 8.1 Hz, 1H), 7.49 – 7.42 (m, 2H), 7.13 (d, J = 7.5 Hz, 1H), 5.42 (d, J = 12.1 Hz, 3H), 5.37 (t, J = 5.9 Hz, 1H), 4.24 (t, J = 6.7 Hz, 1H), 4.19 (d, J = 10.4 Hz, 3H), 4.08 (q, J = 7.1 Hz, 2H), 4.04 – 4.00 (m, 3H), 3.99 (s, 2H), 3.92 – 3.86 (m, 3H), 3.65 (dd, J = 12.0, 8.3 Hz, 3H), 3.38 – 3.22 (m, 4H), 3.05 (dd, J = 10.6, 5.4 Hz, 2H), 2.82 (s, 6H), 2.56 (t, J = 3.2 Hz, 4H), 2.48 (t, J = 10.1 Hz, 3H), 2.45 – 2.32 (m, 6H), 2.28 – 2.25 (m, 1H), 2.25 (d, J = 1.7 Hz, 1H), 2.24 – 2.22 (m, 1H), 2.21 (s, 1H), 2.19 (s, 2H), 2.07 (d, J = 4.0 Hz, 2H), 2.05 (d, J = 4.0 Hz, 1H), 1.96 (d, J = 8.2 Hz, 3H), 1.88 – 1.82 (m, 3H), 1.78 (ddd, J = 9.4, 7.9, 2.8 Hz, 4H), 1.75 – 1.69 (m, 4H), 1.68 – 1.63 (m, 4H), 1.62 (s, 4H), 1.42 – 1.34 (m, 3H), 1.19 (s, 1H), 1.18 (s, 3H), 1.17 (s, 1H), 1.11 (s, 8H), 1.08 (s, 6H), 1.07 (s, 5H), 0.93 (s, 8H), 0.91 (s, 1H), 0.89 (t, J = 7.4 Hz, 1H); ^{13}C NMR (150 MHz, $CDCl_3$) δ 224.4 (C), 212.5 (C), 172.6 (C), 172.5 (C), 152.1 (C), 135.2 (C), 131.1 (C), 135.1 (C), 131.1 (C), 130.5 (C), 130.0 (CH), 129.7 (C), 129.5 (CH), 129.0 (C), 128.5 (CH), 123.4 (CH), 119.0 (CH), 115.4 (CH), 95.3 (C), 73.6 (CH), 69.4 (CH_2), 69.0 (CH_2), 65.7 (C), 65.2 (CH_2), 63.5 (CH_2),

61.1 (CH), 61.0 (CH_2), 59.8 (C), 50.2 (CH), 48.6 (C), 48.1 (CH), 45.6 (CH_3), 43.1 (CH_2), 38.4 (CH_2), 35.9 (CH_2), 33.0 (C), 31.2 (CH), 30.7 (C), 30.6 (CH_3), 29.9 (C), 29.2 (CH_2), 29.1 (CH_2), 27.0 (CH_2), 23.4 (CH_3), 19.3 (C), 19.0 (CH_2), 18.3 (CH_2), 14.3 (CH_3), 11.2 (CH_3); HR-EI-MS (*m/z*): 768.3286 $[M]^+$ (calcd. for $C_{40}H_{52}N_2O_{11}S$, 768.3292).

Preparation of 6-*O*-biotinyl Eriocalyxin B (**EBB8**): To a solution of biotin (18 mg) and DMAP (3 mg) in DMF (1.0 mL) at 0 °C was added DCC (22 mg) in one portion. The mixture was stirred at room temperature for 1 hour. Then compound **1** (17 mg) was added to the mixture. The resulting solution was stirred at room temperature for another 24 hours. And H_2O was added to quench the reaction. The aqueous phase was extracted with EtOAc (10 mL×3). The combined organic layers were washed with brine, dried over anhydrous Na_2SO_4 and concentrated. The crude product was subjected to flash chromatography on silica gel (Chloroform: MeOH = 30 : 1) to give **EBB8** (20 mg, 76% brsm) as white powder. ^{1}H NMR (400 MHz, $CDCl_3$) δ 6.75 (d, *J* = 12 Hz, 1H), 5.89 (d, *J* = 12 Hz, 1H), 5.86 (d, *J* = 4 Hz, 1H), 5.38 (d, *J* = 8 Hz, 1H), 5.36 (s, 1H), 5.30 (s, 1H), 4.49 (dd, *J* = 8 Hz, 4 Hz, 1H), 4.36 (d, *J* = 8 Hz, 1H), 4.31 (dd, *J* = 8 Hz, 4 Hz, 1H), 4.04 (d, *J* = 12 Hz, 1H), 3.12-3.17 (m, 1H), 3.00 (d, *J* = 12 Hz, 4 Hz, 1H), 2.91 (dd, *J* = 12 Hz, 4 Hz, 1H), 2.76 (d, *J* = 12 Hz, 1H), 2.61-2.64 (m, 1H), 2.50 (t, *J* = 8 Hz, 8 Hz, 1H), 2.48 (d, *J* = 12 Hz, 1H), 2.29 (d, *J* = 12 Hz, 1H), 2.12 (dd, *J* = 12 Hz, 4 Hz, 2H), 2.09 (d, *J* = 4 Hz, 1H), 1.78 (dd, *J* = 16 Hz, 8 Hz, 2H), 1.51 (m, 2H), 1.35-1.42 (m, 2H), 1.24 (s, 6H), 1.15 (s, 3H); ^{13}C-NMR (100 MHz, $CDCl_3$) δ 202.6 (C), 196.5 (C), 176.6 (C), 163.6 (C), 160.1 (CH), 153.0 (C), 127.5 (CH), 115.8 (CH_2), 96.7 (C), 73.7 (CH), 65.3 (CH_2), 61.7 (CH), 60.1 (CH), 57.7 (C), 55.3 (CH), 52.5 (CH_2), 47.3 (CH), 46.5 (C), 40.4 (CH_2), 35.7 (C), 33.7 (CH_2), 33.4 (CH), 29.6 (C), 29.4 (CH), 27.7 (CH_2), 25.5 (CH_2), 24.8 (CH_3), 24.0 (CH_2), 18.8 (CH_2); HR-ESI-MS (*m/z*): 571.2486 $[M+H]^+$ (calcd. for $C_{30}H_{39}N_2O_7S$, 571.2477).

Dansyl tagged EriB dominantly labeled a 50-kDa protein

The cytoplasmic fraction and the nucleic fraction of SMMC-7721 cell lysates were incubated with EBF6 (5 μM), with or without the presence of EriB, followed by resolving with SDS-PAGE and imaging under UV transllumitor (Figure S3).

EriB selectively binds to p50 in HEK293T cells

HEK293T cell lysates pretreated with or without TNF-α were incubated with EBB8 in the absence or presence of EriB, or with biotin alone, followed by pull-down with streptavidin beads. The precipitates were resolved by SDS-PAGE, and the gel subjected to immunoblotting with p50 antibody, with β-actin as the input.

Liquid chromatography-mass spectra analysis

Liquid chromatography was performed on a nano Acquity UPLC system (Waters Corporation, Milford, USA) connected to a LTQ Orbitrap XL mass spectrometer (Thermo Scientific, Bremen, Germany) equipped with an online nano-electrospray ion source (Michrom Bioresources, Auburn, USA). Peptides were resuspended with 25 μL solvent A (5% acetonitrile, 0.1% formic acid in water). 20 μL peptide solution was loaded onto the Captrap Peptide column (2mm x 0.5mm, Michrom Bioresources, Auburn, USA) at a 20 μL/min flow rate of solvent A for 5 min and then was separated on a Magic C18AQ reverse phase column (100 μm id×15cm, Michrom Bioresources, Auburn, USA) with a linear gradient. Starting from 5% B (90% acetonitrile, 0.1% formic acid in water) to 45% B (in other words, from 95% A to 55% A, the same below) in 70 min. The column flow rate was maintained at 500 nL/min and column temperature was maintained at 35 °C. The electrospray voltage of 1.4 kV versus the inlet of the mass spectrometer was used.

LTQ Orbitrap XL mass spectrometer was operated in the data-dependent mode to switch automatically between MS and MS/MS acquisition. Survey full-scan MS spectra with one microscan (m/z 350–1800) was acquired in the Obitrap with a mass resolution of 100,000 at m/z 400, followed by MS/MS of the eight most-intense peptide ions in the LTQ analyzer. The automatic gain control (AGC) was set to 1000 000 ions, with maximum accumulation times of 500 ms. For MS/MS, we used an isolation window of 2 m/z and the automatic gain control (AGC) of LTQ was set to 20 000 ions, with maximum accumulation time of 120 ms. Single charge state was rejected and dynamic exclusion was used with two microscans in 15s and 30 s exclusion duration. For MS/MS, precursor ions were activated using 35% normalized collision energy at the default activation q of 0.25 and an activation time of 30 ms. The spectrum were recorded with Xcalibur (version 2.0.7) software.

The mass spectra were searched using the Mascot Daemon software (Version 2.3.0, Matrix Science, London, UK) based on the Mascot algorithm. The database used to search was the protein NFKB1 (P19838). The searching parameters were set up as follows: full trypsin (KR) cleavage with two missed cleavage was considered. Oxidation on methionine and Eriocalyxin B of the Cys

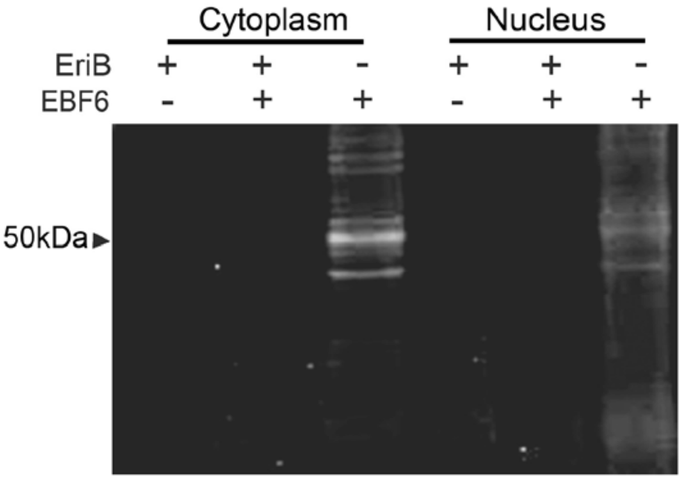

Supplementary Figure S3: Dansyl tagged EriB dominantly labeled a 50-kDa protein.

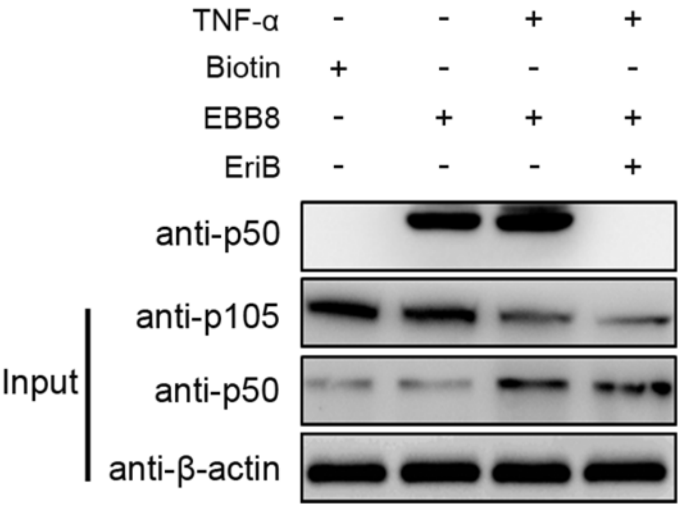

Supplementary Figure S4: EriB selectively binds to p50 in HEK293T cells.

were set as variable modifications. The peptide mass tolerance was 20 ppm and the fragment ion tolerance was 1.0 Da. Peptides whose Mascot expected values below 0.05 were accepted as correct matches.

REFERENCES

1. Niu XM, Li SH, Li ML, Zhao QS, Mei SX, Na Z, Wang SJ, Lin ZW, Sun HD. Cytotoxic ent-kaurane diterpenoids from Isodon eriocalyx var. laxiflora. Planta Medica. 2002; 68:528–533.
2. Zhao Y, Niu XM, Qian LP, Liu ZY, Zhao QS, Sun HD. Synthesis and cytotoxicity of some new eriocalyxin B derivatives. European Journal Of Medicinal Chemistry. 2007; 42:494–502.
3. Wang S, Fang W. Design and synthesis of a dual linker for solid phase synthesis of oleanolic acid derivatives. Molecu les. 2011; 16:4748–4763.
4. Lehtonen, P. Molecular connectivity indexes in the prediction of the retention of oxygen-containing amines in reversed-phase liquid chromatography. J. Chromat ogr. 1987; 398:143–151.
